# Oral Factor Xa Inhibitors versus Warfarin for the Treatment of Venous Thromboembolism in Advanced Chronic Kidney Disease

**DOI:** 10.1155/2021/8870015

**Published:** 2021-01-29

**Authors:** Tania Ahuja, Kelly Sessa, Cristian Merchan, John Papadopoulos, David Green

**Affiliations:** ^1^Medicine, Cardiology & Anticoaguation, NYU Langone Health–Department of Pharmacy, 550 First Avenue, New York, NY 10016, USA; ^2^NYU Langone Health–Department of Pharmacy, 550 First Avenue, New York, NY 10016, USA; ^3^Critical Care & Emergency Medicine, NYU Langone Health-Department of Pharmacy, 550 First Avenue, New York, NY 10016, USA; ^4^Antithrombotic Therapy Team, NYU Langone Health–Department of Medicine, Perlmutter Cancer Center 38th Street, New York, NY 10016, USA

## Abstract

**Introduction:**

Warfarin remains the preferred oral anticoagulant for the treatment of venous thromboembolism (VTE) in patients with advanced chronic kidney disease (CKD). Although the direct oral anticoagulants (DOACs) have become preferred for treatment of VTE in the general population, patients with advanced CKD were excluded from the landmark trials. Postmarketing, safety data have demonstrated oral factor Xa inhibitors (OFXais) such as apixaban and rivaroxaban to be alternatives to warfarin for the prevention of stroke and systemic embolism in patients with atrial fibrillation. However, it remains unknown if these safety data can be extrapolated to the treatment of VTE and CKD.

**Methods:**

A retrospective cohort study from January 2013 to October 2019 was performed at NYU Langone Health. All adult patients with CKD stage 4 or greater, treated with anticoagulation for VTE, were screened. The primary outcome was tolerability of anticoagulant therapy at 3 months, defined as a composite of bleeding, thromboembolic events, and/or discontinuation rates. The secondary outcomes included bleeding, discontinuations, and recurrent thromboembolism.

**Results:**

There were 56 patients evaluated, of which 39 (70%) received warfarin and 17 (30%) received an OFXai (apixaban or rivaroxaban). Tolerability at 3 months was assessed in 48/56 patients (86%). A total of 34/48 (71%) patients tolerated anticoagulation at 3 months, 12 (80%) in the OFXai arm, and 22 (67%) in the warfarin arm (*p*=0.498). There were 10/48 (21%) patients that experienced any bleeding events within 3 months, 7 on warfarin, and 3 on apixaban. Recurrence of thromboembolism within 3 months occurred in 3 patients on warfarin, with no recurrence in the OFXai arm. *Discussion*. OFXais were better tolerated compared to warfarin for the treatment of VTE in CKD, with lower rates of bleeding, discontinuations, and recurrent thromboembolism in a small cohort. Future prospective studies are necessary to confirm these findings.

## 1. Introduction

Chronic kidney disease (CKD) affects more than 37 million patients in the United States [1]. Compared to patients without CKD, patients with CKD stage IV (defined as an estimated glomerular filtration rate (GFR) of 15–30 ml/min) have a two-fold increase in the rates of venous thromboembolism (VTE). The incidence of VTE increases four-fold as compared to the general population, in patients with end-stage renal disease (ESRD), receiving hemodialysis [2]. Physiologically, ESRD results in decreased levels of endogenous anticoagulants and an impaired fibrinolytic system. Despite the increased risk for VTE, patients with CKD stage IV or greater on anticoagulant therapy are more susceptible to bleeding, having a two-time greater risk than the general population [3]. In fact, in one cohort of patients on dialysis, the risk of hemorrhagic stroke was found to be six-fold higher compared to those not on dialysis [3]. The preferred treatment strategy for VTE in the setting of CKD has commonly been warfarin. Alternative treatment regimens to warfarin remain desirable based on the risk of bleeding and warfarin-induced calciphylaxis in patients with ESRD on dialysis [4]. The direct oral anticoagulants (DOACs) have become the preferred treatment strategy for VTE in patients without renal impairment, owing to their noninferiority to warfarin and superiority in risk of bleeding events [5–11]. Although apixaban and rivaroxaban are both FDA approved for use in ESRD with dialysis, these approvals are not based on thromboembolic risks and came after pharmacokinetic studies in healthy volunteers [12–15]. Postmarketing, after this approval, apixaban increasingly became utilized for prevention of stroke and systemic embolism (SSE) in patients with atrial fibrillation (AF) and ESRD given the limited dependence on renal clearance; however, this excluded patients with VTE [13]. In one retrospective cohort, apixaban was found to have a lower incidence of SSE, death, or bleeding event compared to warfarin in those with ESRD and AF [16]. Furthermore, rivaroxaban and apixaban were found to have similar risk of SSE and major bleeding in patients with AF and ESRD receiving dialysis [17]. Although clinicians may be interested in extrapolating from the safety data in the AF population, it remains unknown if apixaban or rivaroxaban, may be used in patients with CKD stage IV or greater for the treatment of VTE. Lastly, the “lead-in” dosing strategies for the treatment of VTE with apixaban during the first week of therapy (10 mg twice daily) and first three weeks of therapy for rivaroxaban (15 mg twice daily) have not been evaluated in patients with CKD/ESRD [7, 16]. Therefore, we aimed to evaluate oral anticoagulant tolerability for the treatment of VTE in patients with CKD stage IV or greater.

## 2. Methods

### 2.1. Study Design

This was a single-center, institutional review board (IRB)-approved, retrospective cohort study at New York University Langone Health (NYULH), an 825-bed academic medical center in New York, NY, from January 2013 through October 2019. Patients were eligible for inclusion if they were ≥18years old, had a diagnosis ICD 9/10 code of VTE plus an estimated glomerular filtration rate (GFR) less than 30 mL/min, and received oral anticoagulant therapy with either warfarin or an oral factor Xa inhibitor (OFXai) including apixaban or rivaroxaban [18]. Electronic health records were further screened to include ICD9 and ICD10 codes for chronic kidney disease (CKD) including ESRD [19–21]. Patients who received less than three doses of an oral anticoagulant and patients with CKD less than IV were excluded.

A retrospective review of the electronic medical record (EMR) was used to obtain baseline demographics and past medical history, including venous thromboembolism (VTE), deep vein thrombosis (DVT), pulmonary embolism (PE), atrial fibrillation (AF), stroke, coronary artery disease (CAD), peripheral vascular disease (PVD), peripheral arterial disease (PAD), heart failure, major surgery or trauma in the past 6 months, gastrointestinal (GI) bleed, smoking in the past year, diabetes mellitus, cirrhosis, coagulopathies, alcohol abuse, active malignancies, and anemia. Other data collected included dosing and duration of anticoagulation, concomitant antiplatelet therapy, presence of a central venous catheter placed during hospitalization, bleeding events, and thromboembolic events. Data were managed utilizing Research Electronic Data Capture (REDCap), a secure informatics system, designed to support data collection across various research disciplines [22].

### 2.2. Outcomes

The primary outcome was tolerability of oral anticoagulant therapy, which was defined as a composite of safety, discontinuation of anticoagulant therapy, and efficacy. Safety was determined by the number of bleeding events, including major, clinically relevant nonmajor, and minor bleeding events within 3 months of initiation of anticoagulation therapy. Major, clinically relevant nonmajor, and minor bleeding was defined using the International Society of Thrombosis and Hemostasis criteria [23]. Reasons for discontinuations included major or minor bleed event, thrombocytopenia, international normalized ratio (INR) testing, cost of therapy, others, or unknown documented reason in the EMR. Secondary outcomes included safety, efficacy, and tolerability as individual endpoints. Efficacy was determined by the number of recurrent thromboembolic events including DVT, PE, or stroke within 3 months of anticoagulation therapy.

### 2.3. Statistical Analysis

Statistical analyses were performed using SPSS version 25 (IBM, Armonk, NY) comparing warfarin to oral factor Xa inhibitors (apixaban/rivaroxaban). Categorical variables were described as frequencies with proportions and compared using a chi-square or Fisher's exact test. Continuous variables were described as medians with interquartile ranges (IQR) and analyzed using Mann–Whitney U. A two-sided alpha of <0.05 was considered to be statistically significant.

## 3. Results

**Figure 1:**
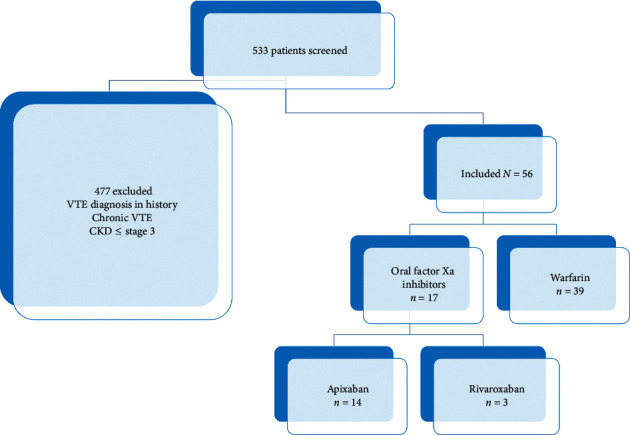
Patient cohort including total number of patients screened and number of patients excluded and included.

A total of 533 patients with an ICD 9/10 code of VTE were screened, and 477 were excluded due to receipt of less than 3 doses of an oral anticoagulant, history of chronic (not acute) VTE, or CKD stage < 4, with 56 included for final analysis (Figure 1). Out of the 56 included, there were 39 (70%) patients that received warfarin and 17 (30%) patients that received an OFXai (*n* = 14 apixaban; *n* = 3 rivaroxaban). The median age of the warfarin group was 71 years (IQR 52, 81) while the median age of the OFXai group was 74 years (IQR 63, 83). More patients in the OFXai group had a history of hypertension (*n* = 17 (100%) vs. *n* = 29 (74%), *p*=0.023) and stage 4 CKD (*n* = 12 (71%) vs. *n* = 14 (36%), *p*=0.017), compared to warfarin, respectively. However, more patients in the warfarin group versus the OFXai group had a history of stage 5 CKD (*n* = 25 (64%) vs. *n* = 5 (30%), *p*=0.017) or were receiving hemodialysis (*n* = 21 (54%) vs. *n* = 4 (24%), *p*=0.036). Of note, more patients in the warfarin group had a decreased EGFR (ml/min/1.73 m^2^) (14 (IQR 7 = 8,23) vs. 21 (IQR 12,28), *p*=0.021) and an increased hospital length of stay (days) (14 (IQR 8,23) vs. 8 (IQR 4,11), *p*=0.006) compared to the OFXai group. In the warfarin and OFXai groups, 19 (49%) and 10 (59%) were on antiplatelet therapy at the time of VTE diagnosis. All other baseline demographics were similar between the two treatment groups (Table 1).

The most common VTE indication for oral anticoagulation therapy was treatment of acute DVT (OFXai *n* = 14 (82%) vs. warfarin *n* = 29 (74%), *p*=0.733) followed by acute PE (OFXai *n* = 4 (24%) vs. warfarin *n* = 10 (26%), *p*=1.000). Lower extremity DVT was more common in both groups compared to upper extremity DVT (OFXai *n* = 11 (65%) vs. warfarin *n* = 20 (51%)). All acute DVTs were diagnosed with a venous duplex ultrasound, and all acute PEs were diagnosed with computed tomography scans. Upon the diagnosis of acute VTE, intravenous unfractionated heparin (UFH) was used in the majority of patients in both arms (OFXai *n* = 10 (59%) vs. warfarin *n* = 36 (92%)) and intravenous argatroban was used in 1 patient in the OFXai group and 1 patient in the warfarin group. The median duration of intravenous UFH was 2.2 days (IQR 0.5, 15) in the apixaban cohort, 2.7 days (IQR 0.2, 6) in the rivaroxaban cohort, and 7.4 days (IQR 2.2, 36.6) in the warfarin group. The median time to oral anticoagulant initiation was 3 days (IQR 0.6, 4) in the apixaban cohort, 6 days (IQR 0.4, 6) in the rivaroxaban cohort, and 2 days (IQR 0.4, 10) in the warfarin group. The remaining ten patients did not receive any intravenous anticoagulant lead-in (*n* = 8 in the apixaban cohort and *n* = 2 in the warfarin cohort). The median “lead-in” doses of OFXai at the time of diagnosis of acute VTE were apixaban 5 mg twice daily (IQR 2.5,10) and rivaroxaban 15 mg twice daily (IQR 5, 15). The median INR percentage in range for warfarin in the 3-month period evaluated was 35% (IQR 25, 52).

### 3.1. Primary Outcome: Tolerability

**Figure 2:**
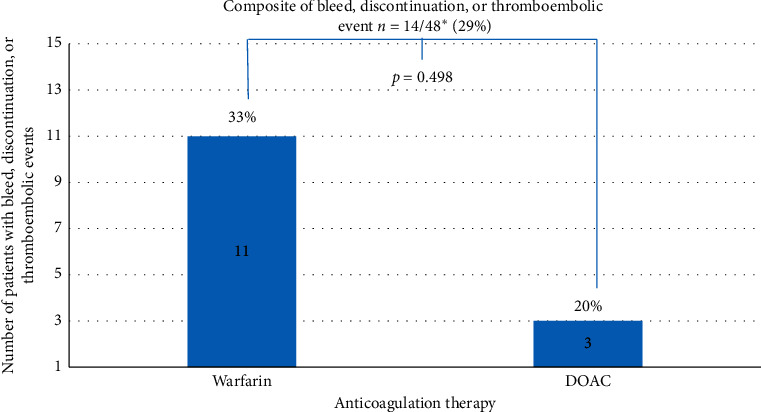
Bar graph of number of patients who had a bleed event, thromboembolic event, and/or discontinued anticoagulation therapy *n* = 14/48 (29%).

Tolerability at 3 months was assessed in 48/56 patients (86%). Six patients were lost to follow-up at 3 months in the warfarin arm, and 2 patients were lost to follow-up in the OFXai arm. A total of 34/48 (71%) patients tolerated anticoagulation therapy at 3 months, 12 (80%) in the OFXai arm, and 22 (67%) in the warfarin arm (*p*=0.498) (Figure 2).

### 3.2. Safety: Bleeding Events

There were 10/48 (21%) patients that experienced any bleeding event within 3 months from anticoagulant initiation (Table 2). The median time to any bleeding event was 39 days (IQR 9, 63) in the total cohort. There was no difference found in major bleeding events between the OFXai and warfarin arms (*p*=1.000). There were 3 (6%) major nonsurgical bleeding events, 5 (10%) clinically relevant nonmajor bleeding events, and 2 (4%) minor bleeding events. Among the patients that experienced a major nonsurgical bleeding event, 1 (6%) occurred while on apixaban and 2 (6%) occurred on warfarin. The bleeding event on apixaban occurred on a dose of 2.5 mg twice daily with no interacting medications identified. The 2 major nonsurgical bleeding events on warfarin occurred in the setting of a supratherapeutic INR, an INR of 4 with gastrointestinal bleed and INR of 7 with psoas hematoma. Out of the 5 clinically relevant nonmajor bleeding events, 1 occurred while on apixaban 5 mg twice daily in the setting of acute on chronic kidney injury and 4 occurred while on warfarin with 3 out of 4 patients with a supratherapeutic INR at the time of bleed. Out of the 2 minor bleeding events, 1 occurred on apixaban at a dose of 2.5 mg twice daily and 1 occurred on warfarin at a therapeutic INR.

### 3.3. Discontinuations of Anticoagulation Therapy

There were 11/48 (23%) patients that discontinued anticoagulation therapy within the 3-month follow-up period (*n* = 3 OFXai and *n* = 8 warfarin). Out of the 3 OFXai discontinuations, all occurred due to bleeding events on apixaban. Out of the 8 warfarin discontinuations, 7 were due to bleeding events and 1 was due to patient-clinician preference. We did not observe any discontinuations in our cohort due to cost or need for laboratory monitoring of warfarin.

### 3.4. Efficacy: Thromboembolic Events

Recurrence of thromboembolism was rare while on anticoagulation therapy, with only 3/48 (6%) patients experiencing a recurrent thromboembolic event at a median of 26 days (IQR 25, 26) from diagnosis of acute VTE. All 3 patients with recurrence (2 DVT, 1 ischemic stroke) occurred while on warfarin at the time of the event, with no recurrence in the OFXai arm at 3 months (*p*=0.542).

## 4. Discussion

We evaluated the tolerability of oral anticoagulants for the treatment of acute VTE in patients with CKD stage IV or greater in this small retrospective cohort. The net clinical benefit of oral anticoagulation for the treatment of acute VTE in those with stage IV CKD or greater remains unknown. We found modest tolerability at 71%, with 14/48 patients either discontinuing anticoagulation therapy, experiencing a bleeding event, or having a recurrent thromboembolic event within 3 months of diagnosis of VTE. Although not statistically significant, given our limited sample size, we observed greater tolerability with OFXai as compared to warfarin for the treatment of acute VTE in those with CKD stage IV or greater, with less bleeding events and no recurrent thromboembolic events at 3 months.

Warfarin remains the preferred anticoagulant in the setting of advanced CKD, given the lack of dependence on renal elimination. However, the narrow therapeutic index, drug-drug interactions, drug-food interactions, and need for laboratory monitoring make alternatives such as the OFXai attractive options to evaluate. Furthermore, the net clinical benefit of oral anticoagulants including warfarin or apixaban in those with advanced CKD has come into question, specifically in the setting of stroke prevention with atrial fibrillation (AF) [24–28]. In one meta-analysis, oral anticoagulants, including warfarin, dabigatran, and rivaroxaban were not associated with a reduced risk of thromboembolism and were associated with an increased risk of bleeding in patients with AF and CKD [28]. There remains a paucity of data evaluating anticoagulation therapy for treatment of acute VTE in the setting of advanced CKD stage IV or greater. In one retrospective cohort of patients with a creatinine clearance of less than 25 mL/min, similar rates of bleeding and thromboembolic events were observed when comparing apixaban to warfarin [29]. As apixaban has limited renal clearance at 27%, there remains interest in evaluating this treatment strategy for acute VTE in the setting of advanced CKD [13]. However, this treatment strategy has not been evaluated in a randomized trial. The risk of thromboembolism within 3 months of diagnosis of an acute VTE may be greater than those placed on oral anticoagulation to decrease the risk of SSE in the setting of AF. Therefore, evaluating all options would be worthwhile to evaluate the most optimal anticoagulant tolerated. As the landmark DOAC trials excluded patients with CKD stage IV or greater, small retrospective cohorts such as ours may give insight into which OFXai may be preferred [5–11]. Out of the 17 patients in our OFXai arm, 14/17 were on apixaban, which is likely due to the limited renal clearance and benefit seen in patients with advanced CKD in preventing SSE in the setting of AF [16]. The risk of VTE and bleeding from oral anticoagulants increases with age [30]. The median age of our cohort was 72 years (54, 81), which may have contributed to the modest tolerability. Furthermore, with cardiovascular comorbidities, we observed a high incidence of combination therapy with antiplatelet therapy with 29/56 (51%) on either aspirin, clopidogrel, or ticagrelor. Previous data have found an increased risk of bleeding events in patients on antiplatelet therapy with oral anticoagulants [31]. Although we did not observe triple antithrombotic therapy, the risk of bleeding in CKD stage IV or greater may be higher in patients on concomitant antiplatelet and anticoagulant therapy. Temporary discontinuation of antiplatelet therapy for the duration of anticoagulant therapy for the treatment of VTE should be explored further, similar to strategies seen in the management of acute coronary syndrome [32].

Dosing considerations for OFXai in the setting of an acute VTE, to reduce the risk of recurrent thromboembolism remains unknown. Although the landmark VTE trials for the OFXai evaluated a “lead-in” with apixaban 10 mg twice daily for 1 week and rivaroxaban at 15 mg twice daily for 3 weeks, before going to maintenance dosing, these doses have not been evaluated even in pharmacokinetic simulations for patients with advanced CKD [7–9]. Therefore, clinicians may be interested in evaluating alternatives such as intravenous heparin at the time of diagnosis of VTE. We observed a high incidence (45/56, 81%) of initial intravenous anticoagulants in our cohort, with the majority occurring in the warfarin arm. This is likely due to warfarin's slow onset of action, with a recommendation to “bridge” with new initiation of warfarin for a minimum of 5 days. This need for intravenous heparin upfront likely contributed to the differences we observed in length of hospitalization within the 2 groups, 14 days for warfarin vs. 8 days for OFXai. Future studies should evaluate either a parenteral anticoagulation lead-in or a higher OFXai dose for safety and tolerability in patients with advanced CKD. We observed that 11/17 patients in the OFXai arm received intravenous anticoagulants, however, for a shorter duration than the warfarin group. Alternatives to intravenous unfractionated heparin in the acute phase of treatment for VTE in those with advanced CKD remain limited. Enoxaparin, a low molecular weight heparin, has been studied in CKD stage 5; however, dose adjustments are warranted due to the dependence on renal clearance [33]. Thus evaluating a direct oral strategy with apixaban or rivaroxaban should be explored.

Our study is limited by its retrospective nature, inability to assess compliance for the entire treatment period, small sample size, and need for acute VTE diagnosis with admission to hospital for study inclusion. Despite these limitations, this study highlights a real-world evaluation of oral anticoagulants in patients with advanced CKD.

In conclusion, the preferred oral anticoagulant for the treatment of VTE in patients with CKD stage IV or greater remains unknown. In our small study, we found OFXai, specifically apixaban, to be an acceptable, possibly safer, alternative to warfarin, in those with advanced CKD. This is similar to findings from larger retrospective studies evaluating apixaban compared to warfarin in those with CKD and AF, for the prevention of SSE [16]. However, the optimal dose, need for either a higher lead-in dose, or lead-in with intravenous anticoagulant should be evaluated in a larger cohort.

## Figures and Tables

**Table 1:** Baseline characteristics.

Demographics	Total cohort *n* = 56	Warfarin *n* = 39	DOAC *n* = 17	*p* value
Age, years, median, IQR	72 (54,81)	71 (52,81)	74 (63,83)	0.433
Male sex	26 (46)	16 (41)	10 (59)	0.219
Wt, kg (ABW), median, IQR	72 (58,88)	61 (50,71)	65 (52,74)	0.423
BMI, kg/m [2], median, IQR	25 (22,31)	25 (22,30)	26 (21,33)	0.397
EGFR, ml/min/1.73m2, median, IQR	17 (8,24)	14 (7,23)	21 (12,28)	0.021*∗*
Length of stay, days, median, IQR	11 (7,21)	14 (8,23)	8 (4,11)	0.006*∗*

*Past medical history*				
DVT	8 (14)	6 (15)	2 (12)	1.000
PE	14 (25)	10 (26)	4 (24)	1.000
Stroke	9 (16)	7 (18)	2 (12)	0.707
Ischemic	7 (13)	5 (12.8)	2 (12)	1.000
Hemorrhagic	2 (5)	2 (5)	0 (0)	1.000
Renal disease				
Stage 4	26 (46)	14 (36)	12 (71)	0.017*∗*
Stage 5	30 (54)	25 (64)	5 (30)	0.017*∗*
Hemodialysis	25 (45)	21 (54)	4 (24)	0.036*∗*
CAD	19 (34)	13 (33)	6 (35)	0.887
PAD/PVD	7 (18)	7 (18)	0 (0)	0.088
Liver disease	1 (3)	1 (3)	0 (0)	1.000
Active cancer	4 (7)	1 (3)	3 (18)	0.079
HF	18 (32)	12 (31)	6 (35)	0.739
Reduced EF	8 (14)	5 (42)	3 (50)	0.688
Preserved EF	10 (18)	7 (58)	3 (50)	1.000
DM	23 (41)	16 (41)	7 (41)	0.992
HTN	46 (82)	29 (74)	17 (100)	0.023*∗*
Prior bleeding	4 (7)	2 (5)	2 (12)	0.577
AF	11 (20)	7 (18)	4 (24)	0.719
CHA_2_DS_2_-VASc, median, IQR	5 (4,7), *n* = 11	5 (4,6), *n* = 7	5 (3,7), *n* = 4	0.527
Anemia	20 (36)	16 (41)	4 (24)	0.209
Coagulopathy	2 (5)	2 (5)	0 (0)	1.000
Protein C/S deficiency		1	0	
Lupus anticoagulant		1	0	
Antithrombin deficiency		0	0	
Antiphospholipid antibody		0	0	
Factor V Leiden		0	0	
Major surgery or trauma in the past 6 months	8 (14)	5 (13)	3 (18)	0.688

*Indication for anticoagulation*				
DVT	44 (79)	30 (77)	14 (82)	0.738
PE	14 (25)	10 (26)	4 (24)	1.000
AF	11 (20)	7 (18)	4 (24)	0.719
Mechanical valve	1 (3)	1 (3)	0 (0)	1.00

*Concomitant medications*				
Aspirin	25 (45)	17 (44)	8 (47)	0.810
Clopidogrel	3 (5)	2 (5)	1 (6)	1.000
Ticagrelor	1 (6)	0 (0)	1 (6)	0.304
Darbepoetin alfa	15 (27)	13 (33)	2 (12)	0.114

All data presented as *n* (%) unless otherwise stated, BMI: body mass index, EGFR: estimated glomerular filtration rate, AF: atrial fibrillation, DVT: deep vein thrombosis, PE: pulmonary embolism, CAD: coronary artery disease, PAD: peripheral artery disease, PVD: peripheral vascular disease, DM: diabetes mellitus, HTN: hypertension, CHA_2_DS_2_-VASc: congestive heart failure, hypertension, age, diabetes mellitus, stroke/TIA/TE, vascular disease (prior MI, PAD, or aortic plaque), and sex category.

**Table 2:** Secondary outcomes.

	Total *n* = 48	Warfarin *n* = 33/39	DOAC *n* = 15/17	*p* value
Thromboembolic event at 3 months	3 (6)	3 (6)	0 (0)	0.542
DVT Ischemic stroke	2 (67) 1 (33)	2 (67) 1 (33)	0 (0) 0 (0)	1.000 1.000
Median time to event, days Median time to any thromboembolic event, median, IQR DVT, median, IQR Ischemic stroke, median, IQR	26 (25,26) 26 (25,41) 37 (37,37)	26 (25,26) 26 (25,41) 37 (37,37)	0 (0,0) 0 (0,0) 0 (0,0)	— — —
Bleed at 3 months	10 (21)	7 (21)	3 (20)	1.000
Major nonsurgical	3 (6)	2 (29)	1 (33)	1.000
Clinically relevant nonmajor	5 (10)	4 (57)	1 (33)	1.000
Minor	2 (4)	1 (14)	1 (33)	0.519
Median time to event, days				
Median time to any bleed, median, IQR	38 (9,63)	37 (4,58)	38 (11,38)	0.667
Major nonsurgical, median, IQR	48 (12,82)	31 (3,58)	90 (90,90)	1.000
Clinically relevant nonmajor, median, IQR	25 (9,71)	25 (4,48)	11 (11,11)	0.800
Minor, median, IQR	83 (76,83)	76 (76,76)	38 (38,38)	1.000

All data presented as *n* (%) unless otherwise stated: 1 patient in the warfarin group experienced a hemorrhagic stroke which occurred at 161 days from AC, 1 patient experienced a clinically relevant nonmajor bleeding after 90 days (139) in the warfarin group, 6 patients were lost to follow-up at 3 months in the warfarin arm, and 2 patients were lost to follow-up in the DOAC arm.

## Data Availability

The data used to support the findings of this study are deposited in a repository that can be accessed if necessary and available upon request.

## Additional Points

 Oral factor Xa inhibitors including apixaban and rivaroxaban may be better tolerated than warfarin for the treatment of VTE in patients with CKD stage IV or greater. Bleeding events with oral anticoagulants remain high in patients with CKD stage IV or greater, however, appear to be less common with apixaban or rivaroxaban compared to warfarin. Future prospective studies evaluating treatment strategies for VTE in patients with CKD stage IV or greater are necessary to confirm findings from this small cohort.
